# Telomerase Cajal body protein 1 depletion inhibits telomerase trafficking to telomeres and induces G_1_ cell cycle arrest in A549 cells

**DOI:** 10.3892/ol.2014.2306

**Published:** 2014-07-02

**Authors:** PING YUAN, ZHITIAN WANG, WANG LV, HUI PAN, YUNHAI YANG, XIAOSHUAI YUAN, JIAN HU

**Affiliations:** Department of Thoracic Surgery, First Affiliated Hospital of Zhejiang University, Hangzhou, Zhejiang 310003, P.R. China

**Keywords:** telomerase Cajal body protein 1, telomerase trafficking, antiproliferation, cell cycle arrest

## Abstract

Telomerase Cajal body protein 1 (TCAB1) is a telomerase holoenzyme, which is markedly enriched in Cajal bodies (CBs) and facilitates the recruitment of telomerase to CBs in the S phase of the cell cycle. This recruitment is dependent on TCAB1 binding to a telomerase RNA component. The majority of cancer cells are able to grow indefinitely due to telomerase and its mechanism of trafficking to telomeres. In the present study, a certain level of TCAB1 expression in A549 human lung cells was identified and TCAB1 knockdown exhibited a potent antiproliferative effect on these cells, which was coupled with a decrease in the cell density and activity of the cellular enzymes. In addition, TCAB1-depletion was demonstrated to inhibit telomerase trafficking to telomeres in the A549 cells, leading to subsequent G_1_ cell cycle arrest without inducing apoptotic cell death. Overall, these observations indicated that TCAB1 may be essential for A549 cell proliferation and cell cycle regulation, and may be a potential candidate for the development of a therapeutic target for lung adenocarcinomas.

## Introduction

Approximately 85% of human cancers restore telomeric DNA base pairs, which have been lost, in order to preserve fertility by activating telomerase (1,2). Telomeres are repetitive DNA sequences (TTAGGG)n present at the termini of chromosomes, which prevent chromosomes from fusing with each other or rearranging. Telomerase is a ribonucleoprotein complex that counterbalances telomere loss by elongating telomeric DNA repeats; this maintains the chromosomal integrity and cells divide continuously in the M phase (3,4). In addition, telomerase is significantly associated with tumor size and aggressiveness, as well as genomic instability and prognosis in the majority of human cancers (5–8). Telomerase consists of two critical components, the RNA subunit (human telomerase RNA component; hTERC), which serves as a template for telomere elongation, and a catalytic subunit (human telomerase reverse transcriptase; hTERT), that provides reverse transcriptase activity (9,10). hTERC is hypothesized to be expressed ubiquitously in all tissues (11), whereas hTERT expression significantly correlates with telomerase activity and appears to be a limiting determinant of enzyme function (12,13).

The accumulation of telomerase in the nucleus is mediated by telomerase Cajal body protein 1 (TCAB1), which is a WD40 repeat protein (also denoted as wdr79 and WRAP53). TCAB1 exists in the active telomerase holoenzyme and it has been demonstrated that TCAB1 is required in HeLa and super telomerase (S-T) cells for the trafficking of telomerase to the Cajal bodies (CBs) during the S phase of the cell cycle (14–16), the phase in which telomere DNA is replicated (17). Previous studies have shown that TCAB1 is a constitutive component of CBs, which has been detected within CBs in a panel of cancer and primary cell lines, including: U2OS, osteosarcoma; H1299, lung cancer; HCT116, colon cancer; HeLa-PV, cervical cancer; HEK293, embryonic kidney cancer; MCF-7, mammary epithelial cells; and HDF, human diploid fibroblasts. Knockdown of TCAB1 impairs the growth of these cancer cells and results in mislocalization of telomerase from CBs to the nucleoli, thus inhibiting the ability of telomerase to restore the telomere length. This consequently leads to massive cell death with several morphological changes in certain cultured cancer cells (18–22).

Annually, more than one million individuals succumb to lung cancer worldwide, thus, it is the leading cause of cancer mortality in humans. Non-small cell lung cancer (NSCLC) accounts for 80% of all the lung cancer types, with adenocarcinoma as the major subtype (23). Since 1995, a paradigm shift in the management of advanced-stage NSCLC has occurred and an expanding array of molecular markers are being used to individualize cancer therapy. As a telomerase holoenzyme component in the telomere synthesis pathway, TCAB1 stably associates with the active telomerase enzyme and directs it through CBs to the telomere in the S phase (20). It was hypothesized in the present study that TCAB1 exhibits the same function in A549 lung adenocarcinoma cells; therefore, to validate this hypothesis a preliminary study was conducted. This demonstrated the function of TCAB1 small interfering (si)RNA in the downregulation of TCAB1 expression in proliferation and enzymatic activities in A549 lung adenocarcinoma cells, without altering telomerase activity. Telomerase/telomere miscolocalization and the consequent cell cycle arrest were further investigated, and the results indicated the possibility of TCAB1 as a potential target during lung adenocarcinoma therapy.

## Materials and methods

### Antibodies

The following three antibodies served as the primary antibodies for western blot (WB) analysis: Monoclonal anti-β-actin (A5441; Sigma-Aldrich, St. Louis, MO, USA); anti-TCAB1 (ab99376; Abcam, Cambridge, MA, USA); and hTERT (RabMAb^®^; 1531-1; Abcam). The secondary antibodies used for WB analysis were as follows: Anti-rabbit IgG (#7074; Cell Signaling Technology, Inc., Danvers, MA, USA) and anti-mouse IgG horse radish peroxidase-conjugated (#7076; Cell Signaling Technology, Inc.). For immunofluorescence (IF), the hTERT (RabMAb^®^; 1531-1; Abcam) and anti-telomeric repeat-binding factor (TRF)-2 (4A794; ab-13579; Abcam) antibodies served as the primary antibodies. Goat polyclonal secondary antibodies against rabbit IgG-H&L (fluorescein-isothiocyanate; ab6717; Abcam) and mouse IgG-H&L (cyanine 3; ab97035; Abcam) served as the secondary antibodies.

### Cell culture, siRNA oligonucleotides, plasmids and transfection

A549 cells obtained from the Cell Bank of the Chinese Academy of Sciences (Shanghai, China) were cultured in F-12K (1237832; Gibco-BRL, Carlsbad, CA, USA) with 10% fetal calf serum (1133067; Gibco-BRL) at 37°C in a humidified incubator with an atmosphere of 5% CO_2_. Lipofectamine 2000 (11668-019; Invitrogen Life Technologies, Carlsbad, CA, USA) was used as the transfection reagent. TCAB1 siRNA (sc-93974; Santa Cruz Biotechnology, Inc., Santa Cruz, CA, USA) was used to knock down TCAB1 and control siRNA (sc-37007; Santa Cruz Biotechnology, Inc.) served as the negative control. Diluted siRNA and control siRNA in F-12K at a concentration of 40 nM were remixed and incubated for 30 min prior to each transfection.

### Cell lysis and WB analysis

Protein was extracted according to the standard instructions for the radioimmunoprecipitation assay lysis buffer (P0013B; Beyotime Institute of Biotechnology, Shanghai, China). The concentration was determined using the DC^TM^ protein assay reagents package (Bio-Rad, Hercules, CA, USA). Next, 30 μg of each sample was loaded onto a 12% polyacrylamide gel, which was electrophoresed in electrophoresis buffer (Thermo Fisher Scientific, Rockford, IL, USA) at 60 V for 30 min until the xylene cyanol dye reached the end of the stacking gel produced in-house (double-distilled H_2_O, 3.4 ml; 30% acrylic bicone 0.83 ml, 1M Tris HCL (pH 6.8), 0.63 ml; 10% sodium dodecyl sulfate, 0.05 ml, 10% ammonium persulfate, 0.05 ml and tetramethylethylenediamine, 0.005 ml). The voltage was subsequently increased to 90 V until the xylene cyanol dye was 1 cm from the bottom of the gel. The protein was transferred to a polyvinylidene fluoride membrane (Seebio Biotech, Inc., Shanghai, China) according to the standard instructions and blocked with 3% bovine serum albumin (BSA) [9408-46-8; Sangon Biotech (Shanghai) Co., Ltd., Shanghai, China] in Tris-buffered saline with Tween 20 (TBST) for 1 h prior to incubation with the primary antibody (1:1,000) overnight at 4°C. The membranes were washed with TBST three times and incubated with diluted secondary antibody (1:1,000). Signals were detected using Pierce enhanced chemiluminescence (Pierce Biotechnology, Inc., Rockford, IL, USA) plus Western Blotting Substrate (PI32132; Thermo Fisher Scientific) and the membranes were visualized on a Kodak XBT-1 film (Kodak, Rochester, NY, USA). All reactions were performed in triplicate.

### Reverse transcription-polymerase chain reaction (RT-PCR) analysis

Total RNA was extracted using TRIzol reagent (11596026; Invitrogen Life Technologies) according to the standard instructions. cDNA synthesized from RNA (1 μg) was used as a template for the RT reaction (R001B; Takara Bio, Inc., Shiga, Japan), which was followed by PCR analysis. The primer sequences were as follows: Forward, 5′-ctc cat cct ggc ctc gct gt-3′ and reverse, 5′-gct gtc acc ttc acc gtt cc-3′ for human actin F; and forward, 5′-aac cgt cag gag ccc act ta-3′ and reverse, 5′-gga gac acc gct tgg aac ta-3′ for TCAB1. RT-PCR was performed under the following conditions: 95°C for 5 min, followed by 30 cycles of 95°C for 30 sec, 55–60°C for 30 sec and 72°C for 30 sec, with a final step of 72°C for 10 min. A total of 10 μl PCR products from each sample were loaded onto a 1.0% agarose gel and electrophoresed at 110 V for 30 min in 0.5X TBE electrophoresis buffer (00124374; Thermo Fisher Scientific). The gel was visualized and the images were captured using an ultraviolet (UV) transilluminator (170–8170, Bio-Rad). All reactions were performed in triplicate.

### Telomerase activity assay

A549 cell pellets were subjected to lysis in 300 μl 3-[(3-Cholamidopropyl)dimethylammonio]-1-propanesulfonate lysis buffer (provided in the kit) and protein concentration standardization was performed using the Bio-Rad Protein Assay reagent (Bio-Rad). For each sample, 1.5 μg cell extract was added to the TRAPeze telomerase detection kit assay (S7707; Millipore, Billerica, MA, USA). The gels were stained with GelRed (41003; Biotium, Inc., Hayward, CA, USA) and visualized with a UV transilluminator. Appropriate positive and negative heat-inactivated cell extracts were set up with test samples and all reactions were performed in triplicate.

### Immunofluorescence assay

Cells were fixed in ice-cold 4% (v/v) paraformaldehyde in phosphate-buffered saline (PBS; pH 7.4) for 15 min at room temperature and washed twice with ice-cold PBS. The samples were incubated for 10 min with PBS containing 0.25% Triton X-100 (room temperature) and washed three times with PBS for 5 min each. The samples were immediately incubated with 1% BSA (9408-46-8; Sangon Biotech) in PBS with Tween 20 (PBST) for 30 min for blocking at room temperature, followed by two primary antibodies (1:1,000) diluted in 3% BSA in PBST (w/v). The cells were subsequently incubated with two primary antibodies at 4°C overnight, washed with PBS three times for 5 min each and incubated with the secondary antibodies (1:1,000) in 1% BSA in PBST for 1 h at room temperature in the dark. Finally, the cells were washed three times with PBS for 5 minutes and visualized at room temperature using a Carl Zeiss microscope (Carl Zeiss AG, Jena, Germany). The exposure times between treatments were consistent and the image brightness and contrast were adjusted using Adobe Photoshop (Adobe Systems Inc., San Jose, CA, USA) for presentation.

### Cell cycle and apoptosis analysis

For the cell cycle assay, the cells were collected and washed with PBS. The cell pellets were obtained by centrifugation at 10 × g (Maxi Mix II rocker rotator - M37610-33CN, Thermo Fisher Scientific) and the supernatant was discarded. Next, 1 ml of DNA staining solution (Multisciences Biotech Co., Ltd, China) was added and blended by vortexing (Maxi Mix II rocker rotator - M37610-33CN Thermo Fisher Scientific) for 10 sec. In preparation for flow cytometry, the cells were incubated for 30 min at room temperature in the dark. For the apoptosis assay, the cells were resuspended in 0.5 ml binding buffer (Santa Cruz Biotechnology, Inc.), containing Annexin V (1:50) and 40 ng/sample of propidium iodide (BD Biosciences, Franklin Lakes, NJ, USA) and incubated for 30 min at 37°C in the dark, prior to flow cytometry. At least 10,000 cells were analyzed for each sample and the experiments were repeated three times.

### Densitometry and statistical analysis

ImageJ software (National Institutes of Health, Bethesda, MD, USA) was used to quantify the band intensity. Data are presented as intensities relative to the indicated loading control and as the mean ± standard deviation of at least three independent experiments. Statistical comparisons were performed using Graph Pad Prism software version 5.01 (GraphPad Software, Inc., La Jolla, CA, USA). P<0.05 was considered to indicate a statistically significant difference.

## Results

### TCAB1 siRNA depletes TCAB1 protein without altering hTERT expression

**Figure 1:**
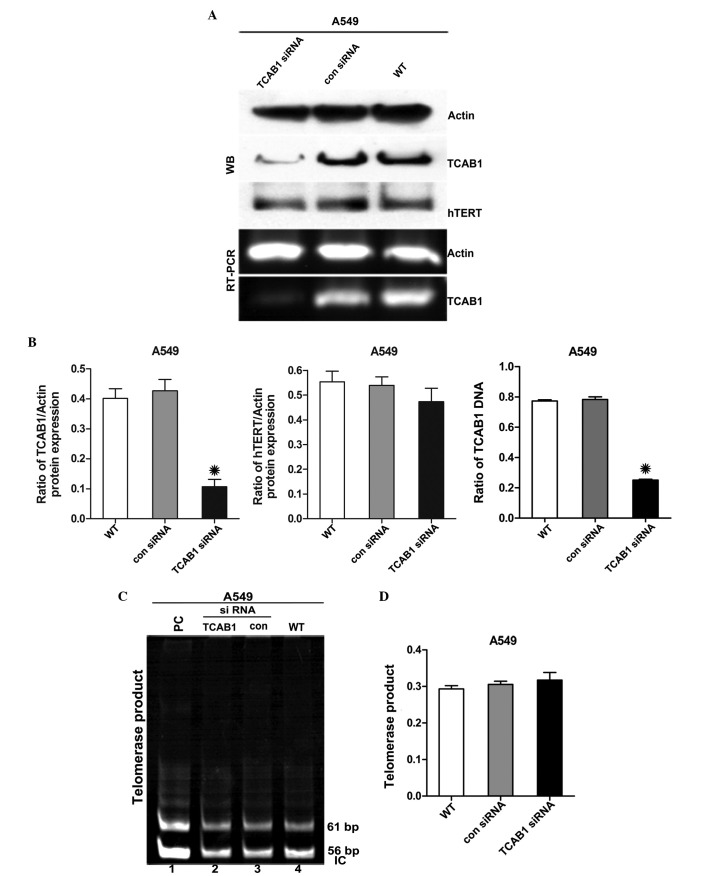
Effect of TCAB1 siRNA on the expression of TCAB1 and hTERT. (A) WB and RT-PCR analyses demonstrate the effect of TCAB1 siRNA transfection on TCAB1 and hTERT expression in A549 cells. (B) Densitometric quantification of the relative TCAB1 and hTERT levels of the cells. Levels of TCAB1 and hTERT were normalized to actin levels. Each bar represents triplicate analyses of the mean ± SD. ^*^P<0.05 vs. con siRNA and WT (n=3). (C) A TRAP assay was performed to evaluate the effect of TCAB1 siRNA treatment on the activity of telomerase in A549 cells. The 56-bp bands represent the IC. (D) The bar graph presents the densitometry-quantified data of the TRAP products in the single lane/PC (line 1) of the TRAP reaction ratios from three independent experiments. Telomerase activities were quantified by comparing the mean band intensity of each lane with the PC. The PC mean band intensity was defined as 100% telomerase-positive. Each bar represents triplicate analyses of the mean ± SD. ^*^P<0.05 vs. con siRNA and WT (n=3). TCAB1, telomerase Cajal body protein 1; con siRNA, control small interfering RNA; WT, wild-type; WB, western blot; hTERT, telomerase reverse transcriptase; RT-PCR, reverse transcription polymerase chain reaction; PC, positive control; IC, internal control; SD, standard deviation; TRAP, telomeric repeat amplification protocol.

TCAB1 expression was investigated in A549 cells. The protein and total RNA were isolated from A549 cells treated with TCAB1 siRNA and control siRNA and the levels of TCAB1 and hTERT were analyzed by WB analysis and RT-PCR. TCAB1 expression was downregulated by ~45% without altering hTERT expression (Fig. 1A and B). Telomeric repeat amplification protocol (TRAP) was also conducted to analyze telomerase activity and the results showed that the telomerase activity remained unchanged compared with the control groups (Fig. 1C).

### TCAB1 silencing inhibits hTERT localization

**Figure 2:**
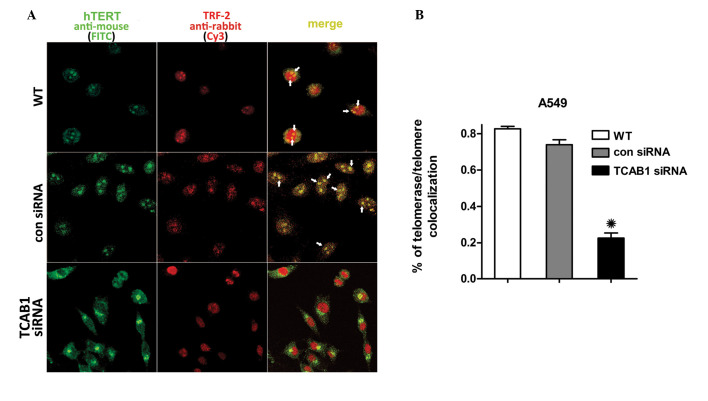
Recruitment of telomerase to the telomere requires TCAB1.(A) Representative hTERT and TRF-2 immunofluorescence data for telomerase and telomeres in A549 cells treated with siRNA against TCAB1. The white arrowheads indicate telomerase/telomere colocalization in A549 cells; the first two rows show foci of telomerase/telomere colocalization in the con siRNA-treated and WT cells. (B) Quantitation of the proportion of telomerase/telomere colocalization among cells from each experiment. The proportion of telomerase/telomere colocalization positivity was calculated by scoring 100 cells from different visual fields from a single treatment and data are presented as the mean ± standard deviation from three independent experiments. ^*^P<0.05 vs. the control (n=3). hTERT, telomerase reverse transcriptase; FITC, fluorescein isothiocyanate; TRF-2, telomere restriction fragment-2; Cy3, cyanine 3; WT, wild-type; con siRNA, control small interfering RNA; TCAB1, telomerase Cajal body protein 1.

The IF revealed that TCAB1 depletion suppressed telomerase trafficking to telomeres in A549 cells. hTERT and TRF-2 are essential subunits of telomerase and telomeres without which they are unable to form. In contrast to the control groups, TCAB1 depletion also suppressed hTERT accumulation at the TRF-2 of the telomeres. The hTERT signal surrounds the membrane of the nucleus in TCAB1-depleted A549 cells, however, does not enter the nucleus, therefore, almost no foci were observed with hTERT/TRF-2 colocalization (Fig. 2A). A total of 100 cells selected randomly from three random fields of each group were scored. hTERT in the cells of the control groups was found to accumulate in the nucleoli and colocalize with TRF-2 more frequently than that in the TCAB1 siRNA-treated cells (Fig. 2B). These results indicated that A549 cells that lack TCAB1 are less likely to transport telomerase to telomeres, which demonstrates the effect of TCAB1 depletion on telomerase recruitment to the telomere.

### Inhibitory effect of TCB1 siRNA on the proliferation of A549 cells

**Figure 3:**
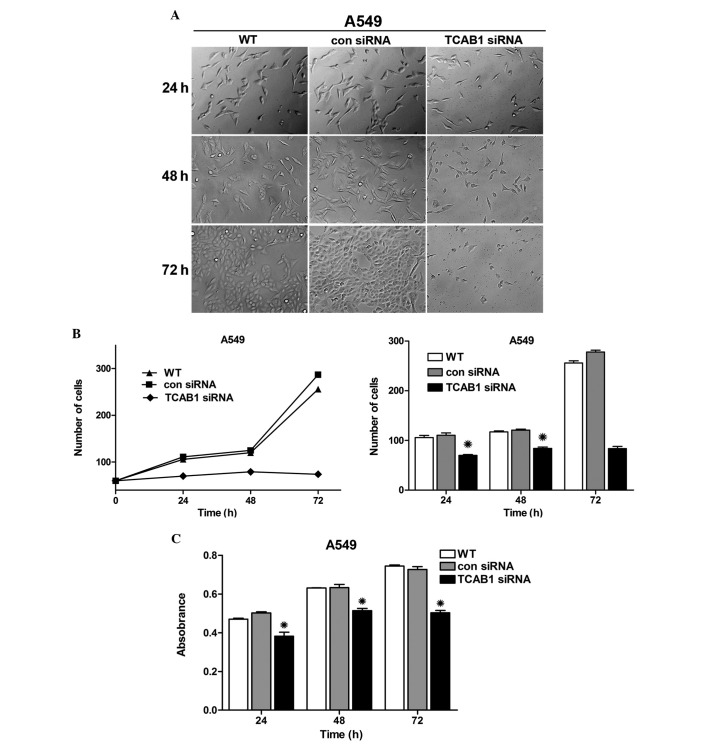
Inhibitory effect of TCAB1 siRNA on the proliferation of A549 cells. (A) Images of selected cells were captured separately at 24, 48 and 72 h (magnification, ×40). Visual fields were selected randomly. (B) The lines reveal differences in cell density as a result of TCAB1 siRNA transfection. The number of cells were counted from three different fields from a single treatment; the line graph shows the overall trends of cell growth, while each bar of the bar graph represents the triplicate counting of each single treatment. Data are presented as the mean ± SD of three experiments. ^*^P<0.05 vs. con siRNA and WT (n=3). (C) MTT assay revealed an evident distinction of the cellular enzyme activities and vitality of the different groups. Data are presented as the mean ± SD of triplicate experiment. ^*^P<0.05 vs. con siRNA and WT (n=3). WT, wild-type; con siRNA, control small interfering RNA; TCAB1, telomerase Cajal body protein 1; SD, standard deviation.

To investigate the impact of TCAB1 depletion in lung adenocarcinoma cells, A549 cells were transfected with TCAB1 siRNA and images were captured using an inverted microscope (magnification, ×40; Olympus Corporation, Tokyo, Japan) separately at 24, 48 and 72 h. Notably, the graphs revealed an evident distinction between cell densities, particularly in the group treated with TCAB1 siRNA (Fig 3A). The graph and bar chart demonstrate a growth trend and cell number difference in each group (Fig 3B), which exhibited an evident weakness in the reproductive capacity of TCAB1-depleted cells. Simultaneously, the activity of cellular enzymes was evaluated by MTT assay and the results were compared to reveal a decreased absorbance in TCAB1-depleted cells, which indicated decreased activity of cellular enzymes (Fig 3C). These results indicated that TCAB1 siRNA effectively inhibits the proliferation of A549 cells.

### TCAB1 depletion arrests the G_1_ cell phase without inducing apoptosis

**Figure 4:**
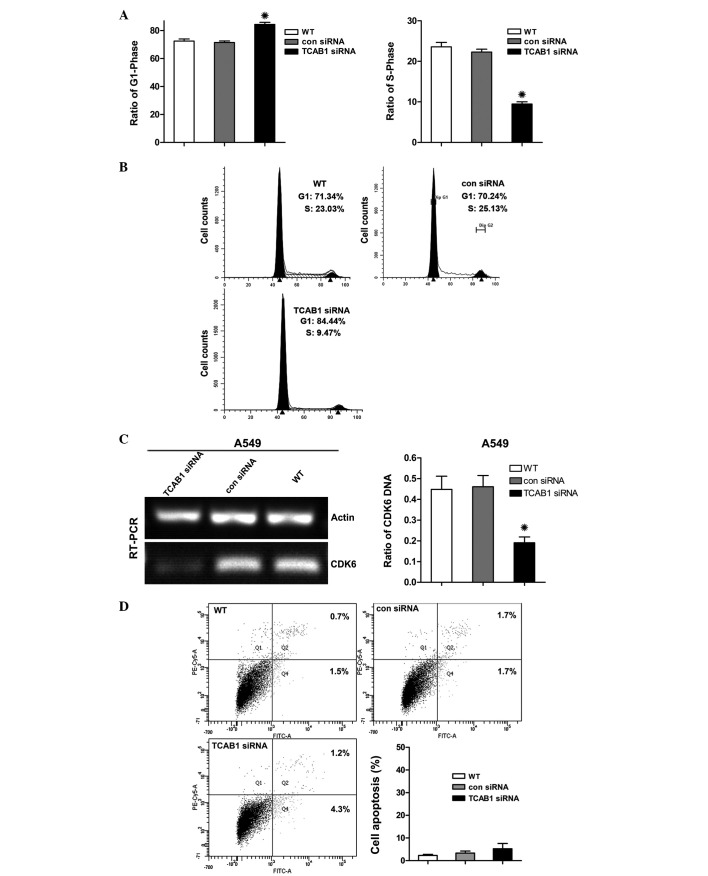
Effect of TCAB1 siRNA on cell cycle and apoptosis regulation. (A) Each bar of the bar graph represents the mean percentage of cells in the G_1_ and S phases. Data are presented as the mean ± SD of triplicate analyses. ^*^P<0.05 vs. con siRNA and WT (n=3). (B) The cell cycle distribution was determined by the detection of DNA degradation. A549 cells were treated with TCAB1 siRNA and stained with PI and analyzed by flow cytometry. The cell count versus PI staining is shown (n=10,000 per treatment). (C) Expression of CDK6 was determined by RT-PCR analysis of the total isolated RNA. Actin served as a gel-loading control. The densitometric quantifications of the CDK6 levels were normalized against the actin levels. Data are presented as the mean ± SD of triplicate analyses. ^*^P<0.05 vs. con siRNA and WT (n=3). (D) Annexin V staining of TCAB1 siRNA-treated cells. Dot-plot data of FITC and PI staining measured by fluorescence-activated cell sorting analysis and graphical representation of apoptosis in cells treated with TCAB1 siRNA. ^*^P<0.05 vs. control siRNA and WT (n=3). WT, wild-type; con siRNA, control small interfering RNA; TCAB1, telomerase Cajal body protein 1; RT-PCR, reverse transcription-polymerase chain reaction; CDK6, cyclin-dependent kinase 6; FITC, fluorescein isothiocyanate; PI, propidium iodide; SD, standard deviation.

Further study was conducted to elucidate the mechanism of action for the antiproliferative activity of TCAB1 depletion on A549 cells. Flow cytometry was used to analyze the A549 cell cycle distribution and apoptosis. Cancer cells are generally immortal and divide uncontrollably, and the replication of telomeres occurs in the S phase (24). Thus, it was predicted that the number of A549 cells would increase in the G_1_ phase and decrease in the S phase as a result of TCAB1 depletion. Consistent with this hypothesis, TCAB1 siRNA was found to induce G_1_ cell cycle arrest, and the percentage of cells with G_1_ DNA content increased from 70 to 88% and the percentage of cells in the S phase decreased from 25 to 9%, when compared with that of the control groups (Fig. 4A). However, cells were not found to accumulate in the sub G_1_ peak (Fig. 4B). In addition, the G_1_ cell cycle checkpoint protein, cyclin-dependent kinase 6 (CDK6), was suppressed (Fig. 4C). Accordingly, the Annexin V staining apoptosis assay of A549 cells treated with TCAB1 siRNA demonstrated no evidence of apoptotic cell death (Fig. 4D). These observations indicated that the antiproliferative effects of TCAB1 siRNA in A549 cells is caused by cell cycle arrest without the induction of apoptosis.

## Discussion

Up to 90% of cancers exhibit activated telomerase that permits cell immortalization and leads to tumorigenesis. Furthermore, the localization of telomerase in CBs in the S phase of the cell cycle is an indispensable step in telomere synthesis. TCAB1 is required in this step in HeLa and S-T cells, however, its depletion has not previously been investigated in A549 lung adenocarcinoma cells. In this initial study, the expression of TCAB1 in TCAB1 siRNA treated and untreated A549 cells was examined and was further integrated with the results regarding TCAB1 expression in NSCLC cell lines. As predicted, TCAB1 was present in the A549 cell line and was downregulated by transfection with TCAB1 siRNA. A previous study has identified that TCAB1 is required for the delivery of telomerase to the nucleus for telomere replication in certain human cancer cells, and that TCAB1 knockdown impairs the growth of these cancer cells (20). However, to evaluate the efficacy and to further elucidate the mechanism of TCAB1 in A549 cells, the current study investigated telomerase/telomere colocalization in TCAB1-depleted A549 cells. IF demonstrated that the hTERT (telomerase) adheres to the membrane of the nucleus and fail to associate with the TRF-2 (telomere) in TCAB1 siRNA-treated cells. Consistently, the graphs of cell density demonstrated that TCAB1 depletion exhibits a potent antiproliferative effect on A549 cells, in addition to decreasing the activity of cellular enzymes in the MTT assay. This antiproliferative effect may be due to the telomerase/telomere miscolocalization caused by TCAB1 depletion. In addition, recent studies have revealed that telomerase (hTERT) depletion in mouse lymphomas results in the emergence of the alternative lengthening of telomere (ALT) (25). ALT is a telomere maintenance mechanism, which exists in telomerase-negative neoplastic and non-neoplastic human cells, characterized by homologous recombination (26–28). The ALT mechanism exists in NSCLC and thus, leads to aggressive malignant properties and the acquisition of resistant mechanisms to counteract telomerase deficiency in late generations of tumor cells (25,29). The association between TCAB1 and telomerase is not fully understood and therefore, to exclude the possibility of telomerase alternation and emergence of ALT in TCAB1-depleted A549 cells, the current study identified the disassociation between TCAB1 depletion and telomerase activity in A549 cells by WB and TRAP. Consistent with the unaltered hTERT expression identified by WB analysis, the results of TRAP also revealed unaltered telomerase activity. Furthermore, the considerable infertility of TCAB1-depleted A549 cells showed no sign of activating the ALT mechanism. These results are consistent with with a study reporting that TCAB1 only functions as a telomerase holoenzyme component in the telomere synthesis pathway following the assembly of the telomerase complex, which contains TERT, TERC and dyskerin (20).

Based on the potent antiproliferative activity, the current study investigated the mechanism of TCAB1 siRNA treatment in the regulation of cell proliferation in A549 cells. Generally, cell cycle arrest and apoptosis are the most common causes of antiproliferation of cells and thus, whether TCAB1-depletion regulates cell cycle progression or induces cellar apoptosis was also investigated. Notably, the cell cycle assay revealed that a greater percentage of cells remain in the G_1_ phase, with a reduced percentage of cells in the S phase following TCAB1 siRNA treatment. However, the sub-G_1_ peak, which is indicative of apoptotic cell death, was not detected. Accordingly, Annexin V staining of TCAB1-depleted cells revealed no evidence of apoptotic cell death. Additionally, G_1_-acting CDK6 expression was investigated and found to be significantly reduced in response to TCAB1 siRNA treatment, indicating that the downregulation of CDK6 may result in the suppression of the cyclin D1-CDK6 complex, which eventually leads to G_1_ phase arrest. These results indicate that the inhibitory effect of TCAB1 depletion on the proliferation of A549 cells is evoked by cell cycle arrest at the G_1_ phase without causing evident apoptotic cell death.

The cell cycle is a process that involves DNA replication and cell division (30). DNA replication occurs during the S phase, and division occurs in the G_2_ and M phases; arrest in any of the phases may inhibit cell proliferation. Furthermore, metabolic activities of DNA at the chromosome ends, where telomeres exist, are important throughout cell cycle progression (31). Telomeric DNA is arranged into folded structures in the G_1_ phase, which unfold to replicate during the process of DNA replication following the recruitment of telomerase to the telomere in the S phase (32). Thus, the present study identified that the infertility of TCAB1 siRNA-treated A549 cells is evoked by G_1_ phase arrest due to TCAB1 depletion, which reduces the presence of telomerase at the telomeres (shown by IF) in the S phase when telomere DNA replication occurs.

In conclusion, the results of the present study demonstrated the function of TCAB1 depletion via decreased telomerase/telomere colocalization proportions, infertility, and reduced activity of cellular enzymes in A549 cells, without downregulating telomerase expression and activity. In addition, it was found that the antiproliferative effect of TCAB1 depletion is evoked by G_1_ phase cell cycle arrest without inducing apoptotic cell death. These results highlight the essential function of TCAB1 within A549 cells. However, the function of TCAB1 in the recruitment of the telomerase complex from the cytoplasm to CBs and restoration of telomere length remains unclear. Recent studies have identified TCAB1 as an essential factor for CBs maintenance and that TCAB1 knockdown prevents the formation of novel CBs (18). Stern *et al* (33) revealed that depletion of CBs also reduces telomerase foci at telomeres. These observations further establish TCAB1 as a significant factor for telomerase recruitment and telomere synthesis. Another study has revealed that the oligonucleotide-binding-fold domain of the telomere-binding protein, TPP1, recruits telomerase to the telomeres through a TERT-associated mechanism (34). The manner in which TCAB1 acts as a cofactor in this mechanism for telomerase trafficking and their connection, as well as the telomere length response in TCAB1-depleted A549 cells, remain to be identified and require further investigation.

Briefly, ‘oncogene addiction’ is defined as the dependence of a cancer cell on one pathway or overactive gene for its survival and/or growth, which provides cancer-specific weaknesses that can be targeted during anticancer therapy (35). In the present study, such properties were identified with regard to the TCAB1 protein. Specifically, TCAB1 is a potential oncogene that is essential for A549 cell survival and, thus, is a notable target for therapeutic intervention in lung adenocarcinoma.

## References

[1] Greider CW, Blackburn EH (1985). Identification of a specific telomere terminal transferase activity in Tetrahymena extracts. Cell.

[2] Shay JW, Bacchetti S (1997). A survey of telomerase activity in human cancer. Eur J Cancer.

[3] Cui W, Wylie D, Aslam S (2003). Telomerase-immortalized sheep fibroblasts can be reprogrammed by nuclear transfer to undergo early development. Biol Reprod.

[4] Bolzán AD (2012). Chromosomal aberrations involving telomeres and interstitial telomeric sequences. Mutagenesis.

[5] Granger MP, Wright WE, Shay JW (2002). Telomerase in cancer and aging. Crit Rev Oncol Hematol.

[6] Hoos A, Hepp HH, Kaul S, Ahlert T, Bastert G, Wallwiener D (1998). Telomerase activity correlates with tumor aggressiveness and reflects therapy effect in breast cancer. Int J Cancer.

[7] Yashima K, Milchgrub S, Gollahon LS (1998). Telomerase enzyme activity and RNA expression during the multistage pathogenesis of breast carcinoma. Clin Cancer Res.

[8] Mokbel K, Williams NJ (2000). Telomerase and breast cancer: from diagnosis to therapy. Int J Surg Investig.

[9] Feng J, Funk WD, Wang SS (1995). The RNA component of human telomerase. Science.

[10] Cech TR, Nakamura TM, Lingner J (1997). Telomerase is a true reverse transcriptase. A review. Biochemistry (Mosc).

[11] Avilion AA, Piatyszek MA, Gupta J, Shay JW, Bacchetti S, Greider CW (1996). Human telomerase RNA and telomerase activity in immortal cell lines and tumor tissues. Cancer Res.

[12] Ito H, Kyo S, Kanaya T, Takakura M, Inoue M, Namiki M (1998). Expression of human telomerase subunits and correlation with telomerase activity in urothelial cancer. Clin Cancer Res.

[13] Kyo S, Kanaya T, Takakura M, Tanaka M, Inoue M (1999). Human telomerase reverse transcriptase as a critical determinant of telomerase activity in normal and malignant endometrial tissues. Int J Cancer.

[14] Cristofari G, Adolf E, Reichenbach P (2007). Human telomerase RNA accumulation in Cajal bodies facilitates telomerase recruitment to telomeres and telomere elongation. Mol Cell.

[15] Zhu Y, Tomlinson RL, Lukowiak AA, Terns RM, Terns MP (2004). Telomerase RNA accumulates in Cajal bodies in human cancer cells. Mol Biol Cell.

[16] Tomlinson RL, Abreu EB, Ziegler T (2008). Telomerase reverse transcriptase is required for the localization of telomerase RNA to cajal bodies and telomeres in human cancer cells. Mol Biol Cell.

[17] Arnoult N, Schluth-Bolard C, Letessier A (2010). Replication timing of human telomeres is chromosome arm-specific, influenced by subtelomeric structures and connected to nuclear localization. PLoS Genet.

[18] Mahmoudi S, Henriksson S, Weibrecht I (2010). WRAP53 is essential for Cajal body formation and for targeting the survival of motor neuron complex to Cajal bodies. PLoS Biol.

[19] Tycowski KT, Shu MD, Kukoyi A, Steitz JA (2009). A conserved WD40 protein binds the Cajal body localization signal of scaRNP particles. Mol Cell.

[20] Venteicher AS, Abreu EB, Meng Z (2009). A human telomerase holoenzyme protein required for Cajal body localization and telomere synthesis. Science.

[21] Egan ED, Collins K (2012). An enhanced H/ACA RNP assembly mechanism for human telomerase RNA. Mol Cell Biol.

[22] Smogorzewska A, van Steensel B, Bianchi A (2000). Control of human telomere length by TRF1 and TRF2. Mol Cell Biol.

[23] Herbst RS, Heymach JV, Lippman SM (2008). Lung cancer. N Engl J Med.

[24] Helmstetter CE (1968). DNA synthesis during the division cycle of rapidly growing *Escherichia coli* B/r. J Mol Biol.

[25] Hu J, Hwang SS, Liesa M (2012). Antitelomerase therapy provokes ALT and mitochondrial adaptive mechanisms in cancer. Cell.

[26] Henson JD, Neumann AA, Yeager TR, Reddel RR (2002). Alternative lengthening of telomeres in mammalian cells. Oncogene.

[27] Slatter TL, Tan X, Yuen YC (2012). The alternative lengthening of telomeres pathway may operate in non-neoplastic human cells. J Pathol.

[28] Lundblad V, Blackburn EH (1993). An alternative pathway for yeast telomere maintenance rescues est1-senescence. Cell.

[29] Bryan TM, Englezou A, Dalla-Pozza L, Dunham MA, Reddel RR (1997). Evidence for an alternative mechanism for maintaining telomere length in human tumors and tumor-derived cell lines. Nat Med.

[30] Pajalunga D, Mazzola A, Franchitto A, Puggioni E, Crescenzi M (2008). The logic and regulation of cell cycle exit and reentry. Cell Mol Life Sci.

[31] Cesare AJ, Karlseder J (2012). A three-state model of telomere control over human proliferative boundaries. Curr Opin Cell Biol.

[32] Verdun RE, Karlseder J (2006). The DNA damage machinery and homologous recombination pathway act consecutively to protect human telomeres. Cell.

[33] Stern JL, Zyner KG, Pickett HA, Cohen SB, Bryan TM (2012). Telomerase recruitment requires both TCAB1 and Cajal bodies independently. Mol Cell Biol.

[34] Zhong FL, Batista LF, Freund A, Pech MF, Venteicher AS, Artandi SE (2012). TPP1 OB-fold domain controls telomere maintenance by recruiting telomerase to chromosome ends. Cell.

[35] Schildkraut JM, Goode EL, Clyde MA (2009). Australian Ovarian Cancer Study Group: Single nucleotide polymorphisms in the TP53 region and susceptibility to invasive epithelial ovarian cancer. Cancer Res.

